# Reimagining the Juvenile Justice System Through the Healthy Outcomes from Positive Experiences Framework

**DOI:** 10.3390/ijerph22050782

**Published:** 2025-05-15

**Authors:** Amanda Winn, Kelsey Hannan, Robert Sege, Dina Burstein

**Affiliations:** Institute for Clinical Research and Health Policy Studies, Tufts Medical Center, Boston, MA 02111, USA; kelsey.hannan@tuftsmedicalcenter.org (K.H.); robert.sege@tuftsmedicine.org (R.S.); dina.burstein@tuftsmedicine.org (D.B.)

**Keywords:** PCEs, resilience, HOPE framework

## Abstract

Numerous research studies have documented the significant influence of key types of positive childhood experiences (PCEs) on adult health and wellbeing, even in the presence of adverse childhood experiences (ACEs). Recent studies reveal that almost 87% of justice-impacted youth reported at least one ACE. Connecting youth to PCEs after trauma has occurred has been shown to disrupt the poor health trajectory associated with ACEs. Creating juvenile justice systems that prioritize equitable access to PCEs has the potential to change the life course of system-impacted youth. The HOPE (Healthy Outcomes from Positive Experiences) framework, a research-based, community-driven approach to improving access to the key types of PCEs youth need to thrive, presents a potentially powerful strategy for juvenile justice systems to transform care for system-impacted youth. This manuscript describes this proposed approach.

## 1. Introduction

Positive childhood experiences (PCEs), including nurturing relationships, safe, equitable environments, opportunities for social and civic engagement, and emotional growth, are essential for optimal growth and development. Numerous research studies have documented the significant influence of PCEs on adolescent and adult outcomes, including physical, mental, and behavioral health [1,2,3,4,5,6,7]. There is also increasing evidence that PCEs can mitigate the negative impacts of adverse childhood experiences (ACEs), which are potentially traumatic experiences of childhood maltreatment or household dysfunction [8,9,10,11,12]. Older research has established the link between ACEs and negative health outcomes [13,14,15,16,17,18], and has associated ACEs with later violence perpetration [19,20] and other antisocial behaviors [21].

An extension of ACE research has examined the prevalence of these experiences among youth involved in the juvenile justice system. One of the early studies of this kind found that youth involved in the juvenile justice system were significantly more likely to have experienced one or multiple ACEs than the adults in the original ACE study and that high-risk youth had a higher number of reported ACEs [22]. Additional research has substantiated the association between ACEs and juvenile justice system contact [23], with an estimate of almost 87% of these youth having at least one ACE [24] and being exposed to three ACEs on average prior to first contact with the justice system [25]. ACEs have also been linked to a higher risk of recidivism [26,27,28], higher rates of psychiatric problems [25,26], and the risk of becoming a serious, violent, and chronic offender [29].

However, there is evidence that PCEs are beneficial for justice-involved youth, leading to fewer psychiatric problems [30,31] and lower recidivism [32,33]. One study estimated 7% decreased odds of recidivism for each additional PCE that justice-involved youth experienced and found that PCEs mitigated the negative impact of ACEs [33]. Additional research from a sample of adolescent offenders and non-offenders found that each PCE was associated with 16% lower odds of arrest and 14% lower odds of delinquent behavior [34].

The Healthy Outcomes from Positive Experiences (HOPE) framework translates findings from the science of PCEs into a practical framework that supports strengths-based approaches to working with youth and families [35]. This approach has the potential to transform the juvenile justice system by offering practical recommendations to promote PCEs among these youth, leading to better outcomes.

The structure of the juvenile justice system has often failed to serve the needs of youth. The adolescent brain is malleable to change, and the juvenile justice system can expose youth to more trauma and unproductive social contexts [36,37]. Several frameworks and approaches have been developed to address these needs. A major movement towards a trauma-informed juvenile justice system focuses on screening and assessing trauma symptoms and providing indicated interventions and a safe environment for youth [38,39]. The specific interventions utilized have shown effectiveness in reducing trauma and behavioral symptoms but have not been evaluated for effects on recidivism or violence [40]. The social ecological model works to move away from a risk-based paradigm and focuses on understanding youth within their relationships and social contexts, engaging youth in positive relationships to promote positive development [41]. Positive youth development (PYD) practices similarly recognize the value of relationships and emphasize youth agency in helping them develop self-efficacy and commitment to their communities [42,43].

The HOPE framework recognizes the role that PCEs can play in resilience from adversity and trauma. This paper describes potentially practical and effective applications of the HOPE framework in the setting of the juvenile justice system leading to increased access to PCEs for system-involved youth. While the approaches above utilize a strengths-based lens to support youth, strengthen youth relationships with others, and create safer environments, the HOPE framework adds a specific focus on understanding and expanding youth access to opportunities for meaningful engagement and mattering and opportunities to process negative emotions in constructive and healthy ways.

## 2. Materials and Methods: The HOPE Framework

**Figure 1:**
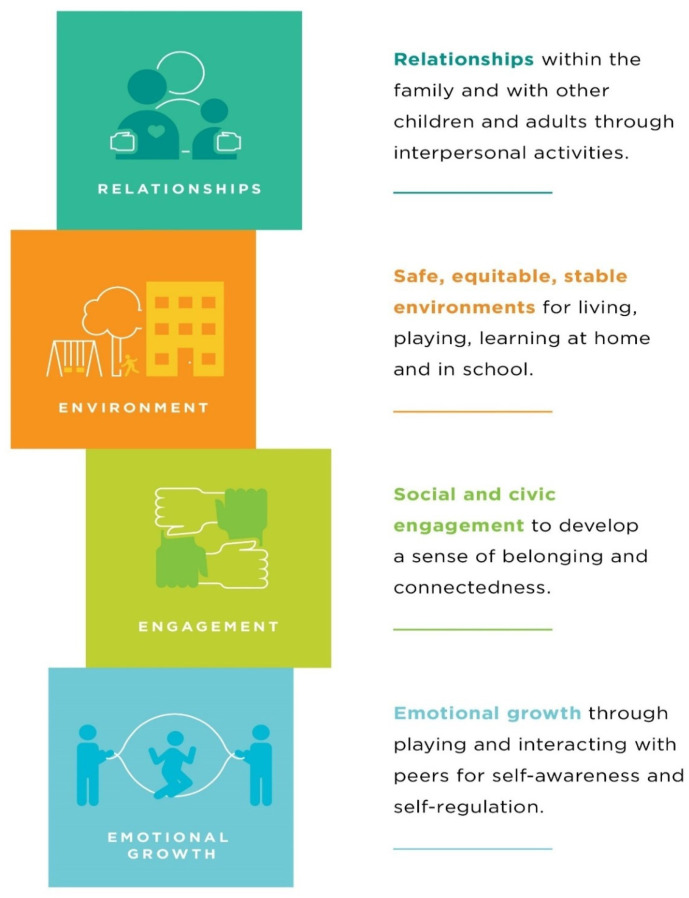
The four building blocks of the HOPE framework.

The HOPE framework is a strengths-based, flexible approach to supporting children and families by prioritizing the promotion of equitable access to the key types of PCEs all youth need to thrive. These key types of PCEs, which HOPE refers to as the four building blocks, were developed by reviewing programs, research, frameworks, and evidence-based models that focused on positive experiences in childhood which led to improved long-term health outcomes. The experiences these programs were delivering clustered around four key areas which grew into the four building blocks [35,44]. The framework is operationalized through a series of strategies and interventions designed to increase access to the four building blocks of HOPE. The HOPE framework helps organizations, communities, and individuals make changes to practices, policies, and programming to ensure that all children have equitable access to PCEs. The four building blocks of HOPE provide an accessible, actionable way of talking about the key types of PCEs that all children need to thrive. The four building blocks (Figure 1) are discussed below.

### 2.1. Relationships

Children and youth need access to safe, supportive, prosocial relationships with caregivers, non-caregiver adults, and peers. Being in nurturing, supportive relationships is critical for children to develop into healthy, resilient adults. Individuals that recall having these types of relationships during childhood experience significantly lower rates of depression, poor mental health, cardiovascular disease, physical health challenges, and substance use during adulthood [1,12,45,46,47,48,49].

### 2.2. Environment

Children and youth who live, learn, and play in safe, stable, and equitable environments have better adult health outcomes [50,51,52]. In addition to meeting the youth’s basic needs, positive environments offer physical and emotional safety for youth.

### 2.3. Engagement

Children and youth need to feel connected to their communities. Involvement in social institutions and organizations, awareness of cultural customs and traditions, and a sense that they matter and belong help youth develop into secure and resilient adults. This development of a sense of mattering and belonging has been shown to have significant impacts on adult health [53,54,55].

### 2.4. Emotional Growth

Children and youth need opportunities to develop their sense of self-awareness and social cognition, learn how to self-regulate emotions and behavior, and acquire skills needed to respond functionally and productively to challenges. Many youths involved in the juvenile justice system have developed unhealthy coping mechanisms and poor regulation skills. Healthy development of these emotional regulation skills is critical for youth to reduce rates of recidivism [56,57].

## 3. Recommendations

Promoting equitable access to the four building blocks of HOPE for justice-involved youth may have long-reaching positive effects. There are many ways professionals in the juvenile justice system can help youth thrive after engagement with the system. Staff can implement HOPE-informed care by identifying, honoring, and promoting positive experiences. The recommendations described below focus on enhancing physical and emotional safety, building meaningful connections, expanding capacity for negative emotions, and empowering both youth and their caregivers. Implementing the HOPE framework within the juvenile justice system has the potential to improve outcomes for system-involved youth.

While approaches such as PYD emphasize building competencies, relationships, and safe environments [42,43,58], HOPE specifically highlights the importance of meaningful engagement and emotional growth as key PCEs in promoting improved outcomes. The emphasis on these additional specific experiences may help system-impacted youth experience a sense of belonging and learn how to manage uncomfortable emotions in positive ways which can mitigate the effects of trauma, help to promote healing, and provide tangible skills to avoid recidivism. HOPE is unique in that it emphasizes the role of four types of PCEs that have the potential to counterbalance the effects of ACEs. In addition, the HOPE framework can be used to shift the juvenile justice system culture, leading to amended policies and practices that focus on creating PCEs for justice system-involved youth. HOPE augments current approaches such as PYD by putting a focus on healing through specific, defined PCEs.

### 3.1. The Intake Process: Asking Specifically About the Four Building Blocks of HOPE

When youth enter the juvenile justice system, there is an opportunity to develop a holistic understanding of that child that can help inform case planning. Asking how system-involved youth currently access the four building blocks provides a systematic structure to identify strengths and challenges. The HOPE framework provides broad categories of PCEs that research shows have profound impacts on adult health and wellbeing. These four building blocks are purposefully general. Learning about individual experiences related to these building blocks offers important insights into each youth’s culture and values. The research clearly shows that safe, supportive relationships are critical for thriving, but each individual determines who those specific people are in their lives. By asking questions at intake about the important people in the youth’s life, how safe they feel at home, at school, and in their community, where they go to find a sense of belonging, and the skills they have to help regulate when they are experiencing strong emotions, the intake worker can collect valuable information that can be used to develop a personalized case plan that seeks to promote healing and resilience for that child. For justice-involved youth, it is also important to understand the dynamics of these relationships and opportunities for engagement, as many youth have found a sense of belonging with peers who also engage in high-risk behaviors. This information, collected at intake, can be crafted into a case plan to increase the youth’s social network and engagement options to include prosocial opportunities.

Obtaining baseline PCE data at intake creates a scenario where progress in this space can be measured over time. Being curious about how youth alter, maintain, or increase access to PCEs while engaged with the juvenile justice system can be a powerful metric of success, both for the youth themselves and for a healing-focused system.

### 3.2. Case Planning: Incorporating the Four Building Blocks of HOPE into Treatment

The juvenile justice system has a unique opportunity to create access to PCEs for some of the most vulnerable and high-risk youth. Case workers can help youth maintain access to existing PCEs and support youth in connecting to the four building blocks in new, meaningful ways. As case workers develop case plans for youth, they can utilize the data obtained from intake to create plans that, in addition to being responsive to court-mandated requirements, also prioritize maintaining and increasing access to the four building blocks. This strategy not only supports healing and resilience for youth, but can also reduce recidivism and subsequent system involvement. Incorporating the four building blocks into case planning will look different for each youth. Some general examples of how to promote each building block are listed below.

Relationships: To strengthen the safe, stable relationships for children involved in the juvenile justice system, providers can ensure youth have access to devices for video communication and establish email or other messaging accounts for safe, confidential correspondence, especially for youth who are in residential treatment programs. When possible, case workers can create specific allowances for youth to spend time with supportive adults. This may include allowing youth pastors, grandparents, or football coaches to visit the youth who are in treatment to help maintain these positive connections while away from home.

Environment: The building block of environment is multi-faceted. Not only do youth deserve access to environments where they feel physically and emotionally secure, but also where they have access to their basic needs. For youth who live with their families, a community resource engagement strategy can be used to connect youth and caregivers to resources within their community, such as housing assistance, food pantries, health clinics, and clothing closets to ensure that those basic needs are being met. Case workers can develop individualized safety plans for managing potential home hazards, such as limiting access to and safe storage of firearms, prescription medications, and other hazardous substances. These approaches also reduce the risk of suicide among youth with affective disorders [59,60]. Youth can be assisted with identifying trusted individuals they could contact if they feel unsafe or are in need of help. For youth in residential treatment programs, case workers should review the processes and protocols the youth can access if they feel unsafe in the program. They can also incorporate individual safety planning for youth who indicate that they have specific needs to help them feel physically and emotionally safe in an out-of-home placement.

Engagement: Developing a sense of mattering and belonging is a key indicator of youth wellbeing. Youth involved with the juvenile justice system often have restricted opportunities for engaging in after-school community activities. When possible, case workers should help them maintain their preferred activity within the treatment facilities. For example, group sports, art, or music activities can promote a sense of belonging and mattering.

For youth who are placed in residential treatment facilities, it is important to keep them connected to their communities. Phone calls with group leaders should be organized so the youth can both stay engaged with the community and retain their group identity. For youth who are not in out-of-home placements, consider creating safety conditions that allow continued participation in group activities in their case plans.

Emotional Growth: The prefrontal cortex of the brain continues to develop into young adulthood [61,62,63]. All youth can benefit from opportunities to learn how to self- or co-regulate, make safe and appropriate decisions, and manage conflict. System-impacted youth who, as mentioned previously, are more likely to have experienced trauma are especially in need of these skills. By understanding from the intake process what tools the youth currently has for managing intense emotions, the case worker can co-create with the youth a case plan that includes meaningful opportunities for emotional growth. Intentional observations of youth behavior can be helpful. For example, a young person who stomps out of the room when frustrated may be successfully re-channeling the impulse to strike another person and on a path towards improved self-regulation. Due to the high incidence of diagnosed mental health conditions, some youth may benefit from behavioral health counseling or parent–child therapy. Strategies for emotional self-regulation and growth often include breathing exercises, yoga, meditation, mindfulness, or spiritual practices. Helping each youth connect with self-regulation practices should be a key component of any case plan.

### 3.3. Screening and Assessments: Using a HOPE-Informed Process to Collect Information

Screenings and assessments, such as substance use screens, mental health assessments, cognitive or intellectual assessments, and ACE screens, can be helpful tools in understanding the specific needs of children, youth, and families. They help staff learn of the challenges young people are facing and allow them to direct their time and energy accordingly. Often, though, obtaining this information can be stressful for both the youth and the provider doing the screening. Whether screening for food and housing insecurity, violence in the home, ACEs, or cognitive ability, there are ways that HOPE-informed providers can conduct these assessments that leave the youth feeling more supported and seen.

Providers can prepare individuals ahead of time by providing information about the screening or assessment that the individual is being asked to complete. It is important for providers to explain the overall goals of the form, the kinds of questions that will be asked, who will have access to the answers, and how the screening results will impact the youth’s case plan. Establishing respect and trust may improve how much the individuals elect to share.

When procedures permit, allowing system-involved youth some control over when and how they share sensitive information may improve the value of standard intake screenings. When the youth is ready to complete the screening or assessment, the provider should begin by reviewing the power of the brain’s capacity to change. This is an appropriate time to review PCEs and the power they have to reduce the physical and mental health outcomes related to ACEs and trauma.

The provider should establish privacy for personal disclosure when possible and create a safe space for the individual to share, acknowledging that there is no pressure to go into detail on any of the questions and reminding them that they can skip any question or stop altogether whenever they would like to.

Once the screening or assessment is complete, the provider should close out the encounter with an intentionally strengths-based message. Report back to the youth what was learned about their positive relationships, environments, engagement, and emotional growth so that they can feel valued. Whatever information was gathered from the assessment will most likely be incorporated into a case plan. Providers should talk openly about what they learned from the tool and how that information may lead to more supportive resources for the youth. Providers can help connect referrals that may come out of screenings and assessments to positive goals. For example, if a mental health assessment revealed that the youth has anxiety and will be referred for treatment and potential medication management, the provider can help the youth understand how managing their anxiety is part of the emotional growth process and will likely lead to them being able to more easily engage in their community, build healthy relationships, and feel safer.

### 3.4. Policies: Ensuring Policies Promote Access to PCEs

The policies that guide daily practice in organizations have the potential to promote or inadvertently block access to the four building blocks. Often, discipline policies in residential treatment facilities and policies related to youth breaking the agreements of their case plan or probation tend to restrict a youth’s access to safe relationships and opportunities for engagement. Restorative justice approaches and engagement in meaningful community service may be more likely to provide opportunities for emotional growth and learning.

The populations involved, resources available, and staffing patterns vary within the juvenile justice system, making it difficult to develop one-size-fits-all behavior management plans. However, intentional focus on the four building blocks of HOPE offers a constructive approach to developing policies that support youth development. Organizations are encouraged to look at policies, especially disciplinary policies or those that are punitive in nature, to understand where there may be opportunities to maintain or even expand youths’ access to PCEs during these learning moments.

## 4. Case Studies

### 4.1. Juvenile Assessment Center

The Community Assessment Program (CAP), a project of the Juvenile Assessment Center in Centennial, CO, USA, strives to keep youth at home and out of juvenile justice and human services systems by connecting families with supportive services to promote safe, healthy, and happy kids. Youth receive early intervention screening and assessment to identify factors contributing to concerning behavior and factors mitigating risks. Referrals are accepted from parents and professionals.

The CAP has incorporated HOPE into the intake, screening/assessment, and case planning process for all youth who receive services in their program. They have added questions about strengths and PCEs into their intake forms. They also specifically assess a child’s community support and strengths during the assessment phase. Case workers create case plans based on the child’s strengths that focus on increasing access to PCEs. The Center believes supporting youth through strengths-based case plans grounded in promoting access to PCEs has been an effective strategy in preventing system involvement for high-risk youth.

### 4.2. Center for Child Counseling

The Center for Child Counseling in Palm Beach Gardens, Florida, is led by a trained HOPE Champion, which is someone who has gone through extensive HOPE training and is certified to guide organizations through the process of HOPE adoption and implementation. The Center had been using PEARLS—Pediatric ACEs and Related Life Events Screener—which screens for adverse childhood experiences as an initial screener for new patients [64]. The Center was struggling with engagement rates, though. Many families would reach out for support, complete the initial intake and screening, and then not return for services. The Center decided to switch to a strengths-based approach in hopes they could shift the experience of any family seeking care and help deal with the “no-show” problem.

The Center now uses the four building blocks of HOPE to guide the interview process. They have shifted their screening process to start with PCEs. This might include asking parents what assets are currently available to the children—whether it be a safe park to play in or a place to get healthy food. It is an opportunity to get information about the things in a child’s environment that influence their wellbeing, and to make them aware of additional resources they might need. By incorporating HOPE into the intake and assessment process, the Center has seen an increase in retention rates and more engagement in the families they work with.

Integrating the HOPE framework into programs for system-involved youth shows promising potential; however, formal evaluation is needed to confirm its effectiveness.

## 5. Conclusions

Research indicates that access to key types of PCEs has long-lasting health and wellbeing impacts well into adulthood. These experiences, understood through the four building blocks of HOPE, are critical for all children, but also serve to promote healing and thriving after trauma for children who have experienced ACEs. Youth who are impacted by the juvenile justice system are likely to have experienced at least one traumatic event. By incorporating the HOPE framework into juvenile justice systems, these vulnerable and high-risk youth may be able to heal and thrive as adults. Promoting access to the four building blocks of HOPE can happen from intake onward in a youth’s juvenile justice journey. The more access these youth have to PCEs, the more likely it is that they will be productive members of their community.

While initiatives such as PYD have provided new strengths-based approaches to work in the juvenile justice system, they may not be fully addressing the needs of these youth. The HOPE framework complements and extends this approach by centering the importance of key PCEs including opportunities for engagement and emotional growth in mitigating the effects of adversity. The HOPE framework has the potential to fill critical gaps that currently exist within juvenile justice reform efforts and may ultimately lead to improved outcomes for system-impacted youth.

The HOPE framework offers a paradigm shift in the understanding of health and wellbeing by emphasizing the importance of positive experiences. The framework provides a blueprint for transforming practice, ensuring the active promotion of access to PCEs. In research, the framework focuses attention on the study of key PCEs in addition to risk factors, and in policy, HOPE fosters the creation of systems that prioritize wellbeing, equity, and opportunity.

## Data Availability

No new data were created or analyzed in this study. Data sharing is not applicable to this article.

## Abbreviations

The following abbreviations are used in this manuscript:

PCEs	Positive childhood experiences
ACEs	Adverse childhood experiences
HOPE	Healthy Outcomes from Positive Experiences
PYD	Positive youth development
CAP	Community Assessment Program
PEARLS	Pediatric ACEs and Related Life Events Screener

## References

[1] Bethell C., Jones J., Gombojav N., Linkenbach J., Sege R. (2019). Positive Childhood Experiences and Adult Mental and Relational Health in a Statewide Sample: Associations Across Adverse Childhood Experiences Levels. JAMA Pediatr..

[2] Crandall A., Broadbent E., Stanfill M., Magnusson B.M., Novilla M.L.B., Hanson C.L., Barnes M.D. (2020). The influence of adverse and advantageous childhood experiences during adolescence on young adult health. Child Abus. Negl..

[3] Graupensperger S., Kilmer J.R., Olson D.C.D., Linkenbach J.W. (2023). Associations Between Positive Childhood Experiences and Adult Smoking and Alcohol Use Behaviors in a Large Statewide Sample. J. Community Health.

[4] Guo S., O’Connor M., Mensah F., Olsson C.A., Goldfeld S., Lacey R.E., Slopen N., Thurber K.A., Priest N. (2022). Measuring Positive Childhood Experiences: Testing the Structural and Predictive Validity of the Health Outcomes From Positive Experiences (HOPE) Framework. Acad. Pediatr..

[5] Huang C.X., Halfon N., Sastry N., Chung P.J., Schickedanz A. (2023). Positive Childhood Experiences and Adult Health Outcomes. Pediatrics.

[6] Slopen N., Chen Y., Guida J.L., Albert M.A., Williams D.R. (2017). Positive childhood experiences and ideal cardiovascular health in midlife: Associations and mediators. Prev. Med..

[7] Tennessee Department of Health Positive Childhood Experiences Among Tennesseans in 2021. https://www.tn.gov/content/dam/tn/health/documents/brfss/PCEs-Factsheet.pdf.

[8] Crandall A., Miller J.R., Cheung A., Novilla L.K., Glade R., Novilla M.L.B., Magnusson B.M., Leavitt B.L., Barnes M.D., Hanson C.L. (2019). ACEs and counter-ACEs: How positive and negative childhood experiences influence adult health. Child Abus. Negl..

[9] Hinojosa M.S., Hinojosa R. (2023). Positive and adverse childhood experiences and mental health outcomes of children. Child Abus. Negl..

[10] Wang D., Jiang Q., Yang Z., Choi J.K. (2021). The longitudinal influences of adverse childhood experiences and positive childhood experiences at family, school, and neighborhood on adolescent depression and anxiety. J. Affect. Disord..

[11] Zhang L., Fang J., Zhang D., Wan Y., Gong C., Su P., Tao F., Sun Y. (2021). Poly-victimization and psychopathological symptoms in adolescence: Examining the potential buffering effect of positive childhood experiences. J. Affect. Disord..

[12] Bellis M.A., Hardcastle K., Ford K., Hughes K., Ashton K., Quigg Z., Butler N. (2017). Does continuous trusted adult support in childhood impart life-course resilience against adverse childhood experiences—A retrospective study on adult health-harming behaviours and mental well-being. BMC Psychiatry.

[13] Felitti V.J., Anda R.F., Nordenberg D., Williamson D.F., Spitz A.M., Edwards V., Koss M.P., Marks J.S. (1998). Relationship of childhood abuse and household dysfunction to many of the leading causes of death in adults. The Adverse Childhood Experiences (ACE) Study. Am. J. Prev. Med..

[14] Bellis M.A., Hughes K., Ford K., Ramos Rodriguez G., Sethi D., Passmore J. (2019). Life course health consequences and associated annual costs of adverse childhood experiences across Europe and North America: A systematic review and meta-analysis. Lancet Public Health.

[15] Bethell C.D., Newacheck P., Hawes E., Halfon N. (2014). Adverse childhood experiences: Assessing the impact on health and school engagement and the mitigating role of resilience. Health Aff..

[16] Merrick M.T., Ford D.C., Ports K.A., Guinn A.S., Chen J., Klevens J., Metzler M., Jones C.M., Simon T.R., Daniel V.M. (2019). Vital Signs: Estimated Proportion of Adult Health Problems Attributable to Adverse Childhood Experiences and Implications for Prevention—25 States, 2015–2017. MMWR Morb. Mortal. Wkly. Rep..

[17] Peterson C., Aslam M.V., Niolon P.H., Bacon S., Bellis M.A., Mercy J.A., Florence C. (2023). Economic burden of health conditions associated with adverse childhood experiences among US adults. JAMA Netw. Open.

[18] Petruccelli K., Davis J., Berman T. (2019). Adverse childhood experiences and associated health outcomes: A systematic review and meta-analysis. Child Abus. Negl..

[19] Duke N.N., Pettingell S.L., McMorris B.J., Borowsky I.W. (2010). Adolescent violence perpetration: Associations with multiple types of adverse childhood experiences. Pediatrics.

[20] Fitton L., Yu R., Fazel S. (2020). Childhood maltreatment and violent outcomes: A systematic review and meta-analysis of prospective studies. Trauma Violence Abus..

[21] Braga T., Goncalves L.C., Basto-Pereira M., Maia A. (2017). Unraveling the link between maltreatment and juvenile antisocial behavior: A meta-analysis of prospective longitudinal studies. Aggress. Violent Behav..

[22] Baglivio M.T., Epps N., Swartz K., Huq M.S., Sheer A., Hardt N.S. (2014). The prevalence of adverse childhood experiences (ACE) in the lives of juvenile offenders. J. Juv. Justice.

[23] Graf G.H.-J., Chihuri S., Blow M., Li G. (2021). Adverse childhood experiences and justice system contact: A systematic review. Pediatrics.

[24] Malvaso C.G., Cale J., Whitten T., Day A., Singh S., Hackett L., Delfabbro P.H., Ross S. (2022). Associations between adverse childhood experiences and trauma among young people who offend: A systematic literature review. Trauma Violence Abus..

[25] Folk J.B., Ramos L., Bath E.P., Rosen B., Marshall B.D., Kemp K., Brown L., Conrad S., Tolou-Shams M. (2021). The prospective impact of adverse childhood experiences on justice-involved youth’s psychiatric symptoms and substance use. J. Consult. Clin. Psychol..

[26] Craig J.M., Zettler H.R., Wolff K.T., Baglivio M.T. (2019). Considering the mediating effects of drug and alcohol use, mental health, and their co-occurrence on the adverse childhood experiences–recidivism relationship. Youth Violence Juv. Justice.

[27] Yohros A. (2023). Examining the relationship between adverse childhood experiences and juvenile recidivism: A systematic review and meta-analysis. Trauma Violence Abus..

[28] Astridge B., Li W.W., McDermott B., Longhitano C. (2023). A systematic review and meta-analysis on adverse childhood experiences: Prevalence in youth offenders and their effects on youth recidivism. Child Abus. Negl..

[29] Fox B.H., Perez N., Cass E., Baglivio M.T., Epps N. (2015). Trauma changes everything: Examining the relationship between adverse childhood experiences and serious, violent and chronic juvenile offenders. Child Abus. Negl..

[30] Clements-Nolle K., Waddington R. (2019). Adverse childhood experiences and psychological distress in juvenile offenders: The protective influence of resilience and youth assets. J. Adolesc. Health.

[31] Logan-Greene P., Tennyson R.L., Nurius P.S., Borja S. (2017). Adverse childhood experiences, coping resources, and mental health problems among court-involved youth. Child Youth Care Forum.

[32] Baglivio M.T., Wolff K.T. (2021). Positive childhood experiences (PCE): Cumulative resiliency in the face of adverse childhood experiences. Youth Violence Juv. Justice.

[33] Kowalski M.A., Hamilton Z., Kigerl A., Baglivio M.T., Wolff K.T. (2023). Protecting against adversity: The role of positive childhood experiences in youth recidivism. Youth Violence Juv. Justice.

[34] Lynne S.D., Fagan A.A., Counts T.M., Bryan J.L., Kidd J., Fogarty K. (2025). Buffering effects of positive childhood experiences on the association between adolescents’ adverse childhood experiences and delinquency: A statewide study. Child Abus. Negl..

[35] Sege R.D., Harper Browne C. (2017). Responding to ACEs With HOPE: Health Outcomes From Positive Experiences. Acad. Pediatr..

[36] Cauffman E., Steinberg L. (2012). Emerging findings from research on adolescent development and juvenile justice. Vict. Offenders.

[37] Cavanagh C. (2022). Healthy adolescent development and the juvenile justice system: Challenges and solutions. Child Dev. Perspect..

[38] Branson C.E., Baetz C.L., Horwitz S.M., Hoagwood K.E. (2017). Trauma-informed juvenile justice systems: A systematic review of definitions and core components. Psychol. Trauma.

[39] Dierkhising C.B., Branson C.E. (2016). Looking Forward: A Research and Policy Agenda for Creating Trauma-Informed Juvenile Justice Systems. J. Juv. Justice.

[40] Zettler H.R. (2021). Much to do about trauma: A systematic review of existing trauma-informed treatments on youth violence and recidivism. Youth Violence Juv. Justice.

[41] Johns D.F., Williams K., Haines K. (2017). Ecological youth justice: Understanding the social ecology of young people’s prolific offending. Youth Justice.

[42] Dillard R., Newman T.J., Kim M. (2019). Promoting youth competence through balanced and restorative justice: A community-based PYD approach. J. Youth Dev..

[43] Butts J.A., Bazemore G., Meroe A.S. (2010). Positive Youth Justice: Framing Justice Interventions Using the Concepts of Positive Youth Development.

[44] Hero J., Gallant L., Burstein D., Newberry S., Qureshi N., Feistel K., Anderson K.N., Hannan K., Sege R. (2025). Health Associations of Positive Childhood Experiences: A Scoping Review of the Literature. Int. J. Environ. Res. Public Health.

[45] Chen P., Harris K.M. (2019). Association of positive family relationships with mental health trajectories from adolescence to midlife. JAMA Pediatr..

[46] Kent B.V., Bradshaw M. (2021). Adolescent Context and Depressive Symptom Trajectories in a National Sample: Ages 13 to 34. Int. J. Ment. Health Addict..

[47] Chopik W.J., Edelstein R.S. (2019). Retrospective memories of parental care and health from mid- to late life. Health Psychol..

[48] Hurd N.M., Stoddard S.A., Bauermeister J.A., Zimmerman M.A. (2014). Natural mentors, mental health, and substance use: Exploring pathways via coping and purpose. Am. J. Orthopsychiatry.

[49] California Department of Public Health, California Department of Social Services, California Essentials for Childhood Initiative, All Children Thrive California (2023). The Adverse and Positive Childhood Experiences Data Report: Behavioral Risk Factor Surveillance System (BRFSS), 2015–2021: An Overview of Adverse and Positive Childhood Experiences.

[50] Steiner R.J., Sheremenko G., Lesesne C., Dittus P.J., Sieving R.E., Ethier K.A. (2019). Adolescent Connectedness and Adult Health Outcomes. Pediatrics.

[51] Parmar D.D., Tabler J., Okumura M.J., Nagata J.M. (2022). Investigating Protective Factors Associated With Mental Health Outcomes in Sexual Minority Youth. J. Adolesc. Health.

[52] Markowitz A.J. (2017). Associations Between School Connection and Depressive Symptoms From Adolescence Through Early Adulthood: Moderation by Early Adversity. J. Res. Adolesc..

[53] Ballard P.J., Hoyt L.T., Pachucki M.C. (2019). Impacts of Adolescent and Young Adult Civic Engagement on Health and Socioeconomic Status in Adulthood. Child Dev..

[54] Porche M.V., Fortuna L.R., Wachholtz A., Stone R.T. (2015). Distal and Proximal Religiosity as Protective Factors for Adolescent and Emerging Adult Alcohol Use. Religions.

[55] Mahoney J.L., Vest A.E. (2012). The Over-Scheduling Hypothesis Revisited: Intensity of Organized Activity Participation During Adolescence and Young Adult Outcomes. J. Res. Adolesc..

[56] Dumornay N.M., Finegold K.E., Chablani A., Elkins L., Krouch S., Baldwin M., Youn S.J., Marques L., Ressler K.J., Moreland-Capuia A. (2022). Improved emotion regulation following a trauma-informed CBT-based intervention associates with reduced risk for recidivism in justice-involved emerging adults. Front. Psychiatry.

[57] Docherty M., Lieman A., Gordon B.L. (2022). Improvement in emotion regulation while detained predicts lower juvenile recidivism. Youth Violence Juv. Justice.

[58] Sanders J., Munford R., Thimasarn-Anwar T., Liebenberg L., Ungar M. (2015). The role of positive youth development practices in building resilience and enhancing wellbeing for at-risk youth. Child Abus. Negl..

[59] Miller M., Azrael D., Barber C. (2012). Suicide mortality in the United States: The importance of attending to method in understanding population-level disparities in the burden of suicide. Annu. Rev. Public Health.

[60] Cai Z., Junus A., Chang Q., Yip P.S.F. (2022). The lethality of suicide methods: A systematic review and meta-analysis. J. Affect. Disord..

[61] Lebel C., Treit S., Beaulieu C. (2019). A review of diffusion MRI of typical white matter development from early childhood to young adulthood. NMR Biomed..

[62] Kolk S.M., Rakic P. (2022). Development of prefrontal cortex. Neuropsychopharmacology.

[63] Teffer K., Semendeferi K. (2012). Human prefrontal cortex: Evolution, development, and pathology. Prog. Brain Res..

[64] Koita K., Long D., Hessler D., Benson M., Daley K., Bucci M., Thakur N., Burke Harris N. (2018). Development and implementation of a pediatric adverse childhood experiences (ACEs) and other determinants of health questionnaire in the pediatric medical home: A pilot study. PLoS ONE.

